# The effects of graded levels of calorie restriction: VIII. Impact of short term calorie and protein restriction on basal metabolic rate in the C57BL/6 mouse

**DOI:** 10.18632/oncotarget.15294

**Published:** 2017-02-11

**Authors:** Sharon E. Mitchell, ZhanHui Tang, Celine Kerbois, Camille Delville, Davina Derous, Cara L. Green, Yingchun Wang, Jackie J.D. Han, Luonan Chen, Alex Douglas, David Lusseau, Daniel E.L. Promislow, John R. Speakman

**Affiliations:** ^1^ Institute of Biological and Environmental Sciences, University of Aberdeen, Aberdeen, Scotland, UK; ^2^ State Key Laboratory of Molecular Developmental Biology, Institute of Genetics and Developmental Biology, Chinese Academy of Sciences, Beijing, China; ^3^ Key Laboratory of Computational Biology, Chinese Academy of Sciences-Max Planck Partner Institute for Computational Biology, Shanghai Institutes for Biological Sciences, Chinese Academy of Sciences, Shanghai, China; ^4^ Key Laboratory of Systems Biology, Innovation Center for Cell Signaling Network, Institute of Biochemistry and Cell Biology, Shanghai Institutes of Biological Sciences, Chinese Academy of Sciences, Shanghai, China; ^5^ Department of Pathology, University of Washington, Seattle, Washington, USA; ^6^ Department of Biology, University of Washington, Seattle, Washington, USA

**Keywords:** metabolic rate, dietary restriction, protein restriction, calorie restriction, metabolic suppression, Gerotarget

## Abstract

Under calorie restriction (CR) animals need to lower energy demands. Whether this involves a reduction in cellular metabolism is an issue of contention. We exposed C57BL/6 mice to graded CR for 3 months, measured BMR and dissected out 20 body compartments. From a separate age-matched group (*n*=57), we built 7 predictive models for BMR. Unadjusted BMR declined with severity of restriction. Comparison of measured and predicted BMR from the simple models suggested suppression occurred. The extent of suppression was greater with increased CR severity. However, when models based on individual organ sizes as predictors were used, the discrepancy between the prediction and the observed BMR disappeared. This suggested metabolic suppression was an artefact of not having a detailed enough model to predict the expected changes in metabolism. Our data have wide implications because they indicate that inferred metabolic impacts of genetic and other manipulations may reflect effects on organ morphology.

## INTRODUCTION

Feeding animals less energy, or calorie restriction (CR) was the first environmental manipulation that was consistently demonstrated to retard the aging process and thereby result in improved healthspan, as well as increased mean and maximum lifespan [1, 2]. The beneficial effects of CR have been demonstrated across a wide range of taxa [3–5]. However, in recent years it has become apparent that the impact of CR on aging is not universal. Different strains of mice appear to react differently when exposed to CR, with some strains actually living shorter lives [6–8]. Moreover, separate cohorts of non-human primates reacted differently to CR in their lifespans, with one group showing increased lifespan but the other not, but in both cases there was improved healthspan [9–11]. These differences were attributed to potential genetic differences between the cohorts or the different diets they were fed [10, 11].

This complexity in the response of animals to CR is coupled with the fact that despite decades of intense interest, in models where we know it has a beneficial impact, the physiological and molecular mechanisms that underpin its action remain elusive. Confusion about the impact of CR is perhaps best exemplified by studies of the effect of CR on metabolic rate. Reduced metabolic rate was, for a long time, presumed to be the causal mechanism underpinning the lifespan extending effects of CR. This is because at the whole animal level, if individuals are to regain energy balance at the new lower level of energy intake, under a CR treatment, they must reduce their overall expenditure of energy to match the level of energy intake. Otherwise they would remain in negative energy balance, and continue losing weight until they eventually starved to death. One mechanism to reduce energy expenditure would be to become less active. Studies however suggest that animals under restriction do not substantially reduce their physical activity levels [12–14]. This may be because being active is the only way in the wild that they can find new sources of food, hence reducing activity might be counter-productive. Nevertheless they may still save energy spent on physical activity because they are smaller and the cost of moving around is reduced. The main other way they can save energy is by reducing their non-activity metabolism or resting metabolic rate (RMR). RMR is defined as the energy demands of an animal at rest. A more rigorously constrained measurement is basal metabolic rate (BMR). This is the measure of the metabolic rate of an animal at rest, that is also post-absorptive, at eurythermic body temperature (i.e. not torpid) and in the thermoneutral zone. Hambly and Speakman (2005) partitioned the savings of energy under 20% CR, and suggested the savings from reduced RMR were about half of that due to the reduced costs of physical activity [15]. Because animals have to obey the laws of thermodynamics, the greater the level of restriction, the larger the reduction in metabolic rate must be to compensate. Since the effect of CR on lifespan is also directly related to the extent of restriction [16] there is a strong positive correlation between the extent of reduction in metabolic rate and the extent of lifespan extension.

However, while the effect of CR on total expenditure of calories must be negative, the expenditure of energy at the tissue level is not so constrained. This is because animals can reduce their RMR by becoming physically smaller. Consequently the tissue level utilization of energy could in theory follow any pattern when animals are under CR. Consider the following theoretical example (from [2]): if an animal was placed on 30% CR, to bring itself into energy balance the animal could uniformly reduce the size of all its organs by 30%, and without any modulation of the cellular rate of energy metabolism it would be back in balance. Alternatively, it may decrease its organs by only 20% and effect the additional 10% reduction in energy utilization by depressing cellular energy use. Or it might reduce the size of its organs by 40%, allowing it to increase expenditure at the tissue level by 10% [2]. These are clearly only theoretical possibilities, but there has been considerable debate about the actual effect CR has on energy expenditure at the tissue level. Many studies have suggested that when adjustments for body mass are made the energy expenditure is not different between CR and *ad libitum* (AL) animals [17–23]. However there are also many studies suggesting that even after adjustment for body mass differences, there is still a reduction in RMR at the tissue level, e.g. [24–27]. This lowered RMR would be consistent with the widely observed lower body temperature in animals under CR [28–31].

It seems differences between studies are at least in part caused by differences in the exact procedures used to normalize for body mass changes [32, 33]. This is not straightforward because the energy expenditure differs across tissues [34] and animals under CR do not lose tissue uniformly [35]. Indeed the different patterns of tissue utilization may be driven by the need to reduce overall energy requirements while retaining organ functionality. Many researchers have recognized the importance of changed body composition under restriction and have attempted to ‘correct’ for this effect in their expressions of metabolic rate [1, 36]. These corrections have been made in two different ways. The first is by expressing the metabolic rate divided by body mass raised to an exponent (normally 0.75 or 0.66). The justification for this approach is that differences in body mass between species scale with a gradient of approximately 0.75. However, the processes that generate the interspecific scaling exponent are likely very different from the differences between individuals within a species [32]. Similar problems attend the use of the other common scaling exponent used for correction of size effects (0.66), which addresses interspecific surface area changes with size. A second common way to correct for body mass changes in CR studies is to express the metabolic rate in relation to the changes in lean body mass. The basis of this argument is that fat tissue has a substantially lower metabolic rate than lean tissue. However, while fat tissue has a substantially lower metabolic rate than lean tissue *in vitro*, the magnitude of the effect *in vivo* is less apparent [37–39]. Yet, expressing metabolism divided by lean body mass makes the assumption that fat tissue contributes nothing to metabolic rates. This can lead to some spurious inferences when large changes in body composition occur [22, 25, 32]. At present it is almost impossible to resolve whether CR results in a reduction, no change or an increase in tissue level metabolic rate in animals at rest.

In an attempt to overcome the issue of changes in body composition on energy demands of rats under CR, Selman *et al* (2005) measured daily energy expenditure (DEE) of both CR (40% starting at 4 months of age) and AL fed Fischer 344 rats at two different ages (6 and 26 months) [40]. DEE was measured using the doubly-labeled water (DLW) method. These were related to a detailed body composition analysis, and using only the animals fed AL a multiple regression analysis was used to link together the individual variability in organ masses with the variation in DEE. This model was then applied to predict the expected DEE of the animals under CR. Actual measurements of the animals under CR were all higher than the prediction suggesting at the tissue level metabolic rate was increased. This study was exceptional, however, in that the dependent variable was the total DEE, rather than the RMR, and changes in the contribution of activity expenditure were unknown, leaving it open that responses in RMR might follow a different pattern.

Over a series of recent papers we have described the diversity of transcriptomic, physiological, endocrine, biochemical and behavioral responses of C57BL/6 mice to graded levels of short term (3 months) CR [14, 31, 35, 41–44]. In some cases these responses have been compared to the equivalent responses to levels of protein restriction (PR) [14, 31, 35, 41]. Here we focus on BMR utilizing an essentially similar approach to that adopted by Selman *et al* (2005) [40]. In short we used an independent group of 58 animals to build a statistical model of how organ masses contribute to metabolic rate, and then applied this model to predict the BMR of 48 mice under varying levels of CR and 32 mice under varying levels of PR, from the previously published detailed data on their body compositions [35]. We then compared the predictions to the actual measurements of BMR in the same animals to establish if there was a suppression of metabolism or not, and its hormonal correlates, assayed also in the same individuals [41].

## RESULTS

### Raw unadjusted BMR

**Figure 1:**
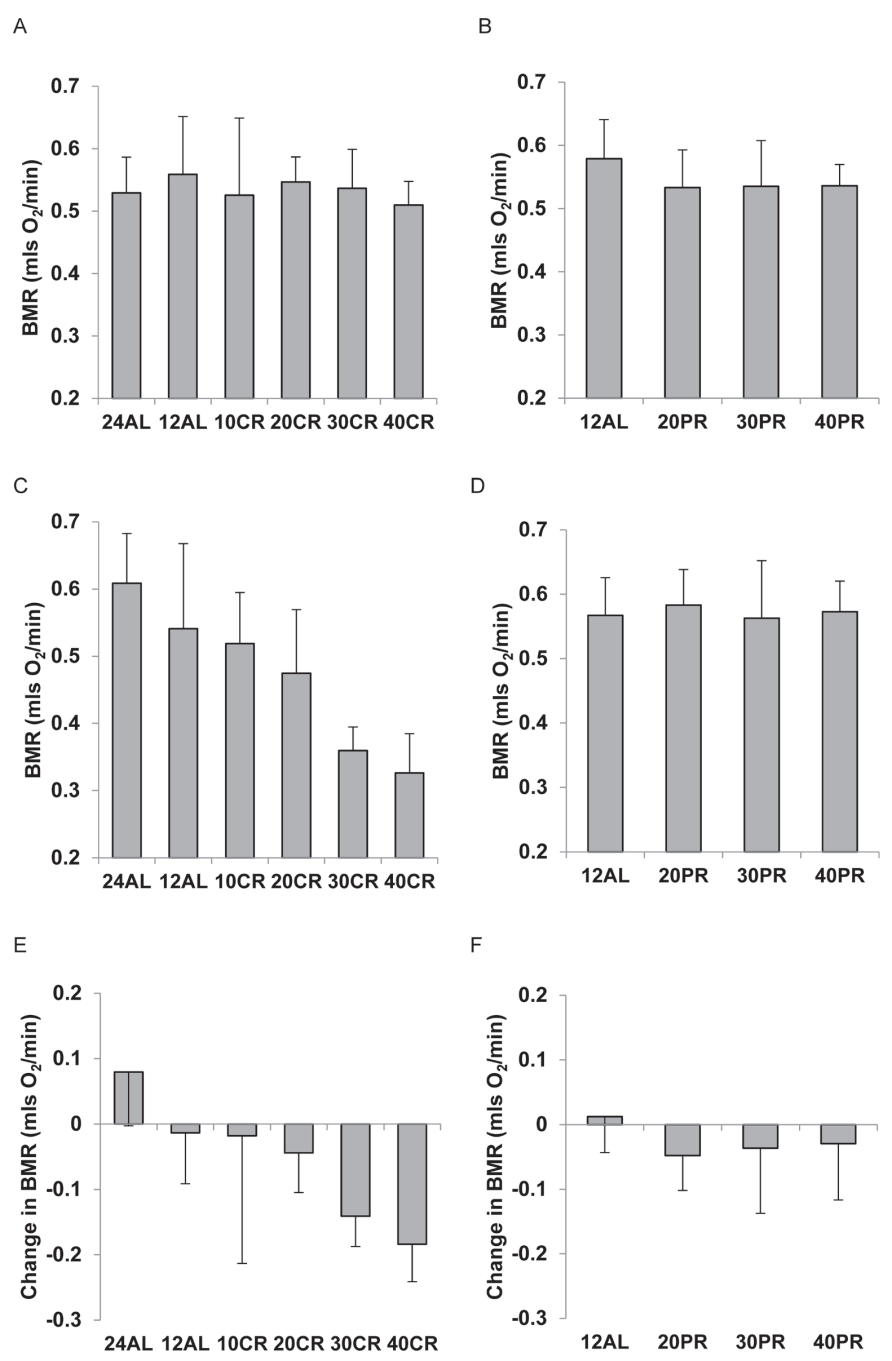
Basal metabolic rate (BMR) (mls O_2_/min) of C57BL/6 mice **A**. at baseline for mice aged 5 months in relation to randomized calorie restriction (CR) grouping and **B**. in relation to randomized protein restriction (PR) grouping. **C**. after 3 months CR, in relation to restriction group and **D**. after 3 months PR, in relation to restriction group. **E**. change in BMR between baseline and the end of restriction (3 months) in relation to CR group and **F**. change in relation to PR group. Mice did not differ prior to randomization but in the CR exposed animals BMR was reduced in direct relation to the extent of restriction. Under equivalent levels of PR no such change was observed. For details of statistics refer to text. 24AL refers to mice with constant access to food.12AL mice had *ad libitum* access for 12h per day. 10CR, 20CR 30CR and 40CR refer to mice under 10, 20, 30 and 40% CR respectively, while 20PR, 30PR and 40PR refer to mice under 20, 30 and 40% PR respectively.

As expected there was no significant difference in the BMR between the treatment groups at baseline prior to them being placed on treatment, for the CR mice (ANOVA F_5,41_ = 0.43, *P* = 0.828: Figure 1A) and for the PR mice (ANOVA F_3,28_ = 1.15, *P* = 0.347: Figure 1B). Following 3 months of CR there was a large significant treatment effect on BMR (ANOVA F_5,41_ = 12.78, *P* < 0.0005: Figure 1C) with a clear progressive lowering of the BMR as the level of CR increased. In fact the BMR of the animals on 40% restriction (40 CR) was only 54% of the BMR of the 24AL group (fed 24 hrs *ad libitum*) (that is 56% lower), and 60.3% of the BMR of the 12AL group (ie 39.7% lower) (both comparisons significant *P* < 0.01 by Tukey post hoc comparison). In contrast, in the PR animals there was no significant difference in the BMR between the different PR groups after 3 months on PR (ANOVA F_3,28_ = 0.15, *P* = 0.929: Figure 1D). There was a strong effect of treatment group on the change in BMR in mice exposed to 3 months of CR (ANOVA F_5,40_ = 7.77, *P* < 0.0005: Figure 1E) showing that BMR had declined by progressively larger amounts as the extent of restriction was increased. That is for the 24AL group the BMR had actually increased by on average 14.2%, it declined by 3.14% on average for the 12AL mice, but declined by 36% for those on 40CR. Contrasting these large declines, for the PR animals there was no significant treatment effect on the difference in BMR between the baseline and final measurements (ANOVA: F_3,28_ = 0.92, *P* = 0.443: Figure 1F).

### BMR adjusted by ratio methods

**Figure 2:**
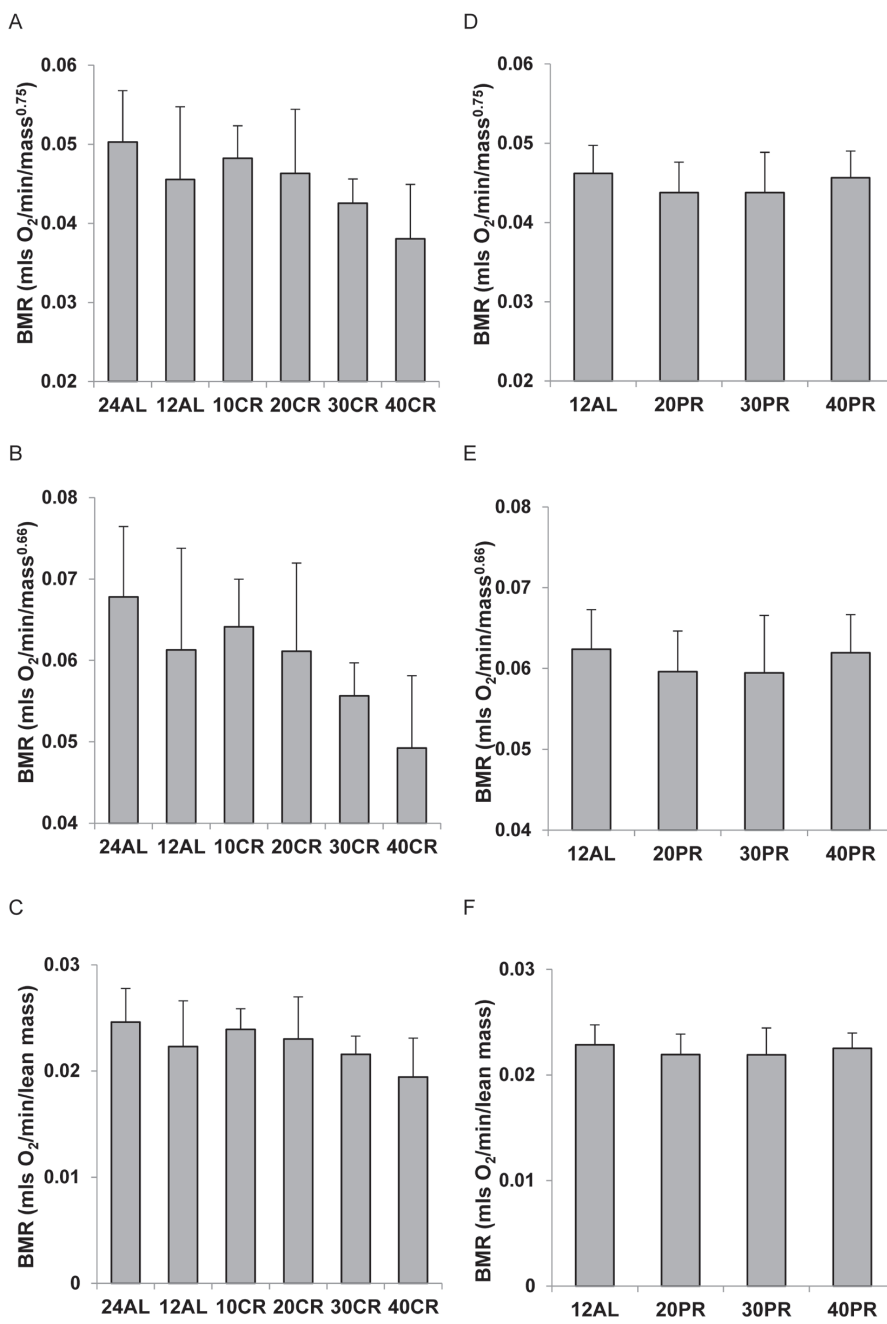
Basal metabolic rate (BMR) of mice after 3 months of calorie (CR) or protein (PR) restriction, normalized using 3 popular ratio methods **A**. CR, and **D**. PR normalized by dividing BMR (mls O_2_/min) by body mass^0.75^. **B**. CR and **E**. PR normalized by dividing BMR (mls O_2_/min) by body mass^0.66^. **C**. CR and **F**. PR normalized by dividing BMR (mls O_2_/min) by lean body mass. In all cases there was a significant effect of CR on the normalized metabolic rate and with PR there was no significant effect. For details of statistics refer to text. 24AL refers to mice with constant access to food.12AL mice had *ad libitum* access for 12h per day. 10CR, 20CR 30CR and 40CR refer to mice under 10, 20, 30 and 40% CR respectively, while 20PR, 30PR and 40PR refer to mice under 20, 30 and 40% PR respectively.

We used three popular ratio based methods to adjust the final measures of BMR for differences in the body mass of the animals at the end of the treatment period. These included dividing BMR by the total body mass (sum of all dissected organs) raised to the 0.66 or 0.75 powers, and dividing BMR by the lean tissue mass. In the CR treated animals whether BMR was adjusted by dividing by body mass^0.75^ or body mass^0.66^, both corrections still showed progressive lowering of the metabolism in relation to the level of restriction (mass^0.75^: ANOVA F_5,41_ = 3.42, *P* = 0.011: Figure 2A and mass^0.66^: ANOVA F_5,41_ = 4.38, *P* = 0.003: Figure 2B). This was also the case when the BMR was adjusted by dividing by lean tissue mass (ANOVA: F_5,41_ = 2.55, *P* = 0.045: Figure 2C). The extent of the declines were, however, lower following adjustment, than for the raw data. Comparing the 24AL group to the 40CR group the average declines were 32.1% when using Mass^0.75^, 37.6% when using Mass^0.66^ and 26.8% when adjusting by lean mass. The equivalent figures compared to the 12AL group were 26.8%, 30.3% and 23.2% respectively. Whichever method of ratio normalization was used, and whichever of the two control groups was used as the comparison, the indication was that there was a progressive suppression of the metabolism, which under 40% CR was in the region of 23.2 to 37.6%. For the mice under PR there was no effect of the treatment on the BMR when it was normalized by mass^0.75^ (ANOVA: F_3,28_ = 0.78, *P* = 0.516: Figure 2D) by mass^0.66^ (ANOVA: F_3,28_ = 0.62, *P* = 0.607: Figure 2E) or by lean mass (ANOVA: F_3,28_ = 0.42, *P* = 0.737: Figure 2F). One interpretation of these data is that basal metabolism was suppressed under CR but equivalent levels of PR did not produce any suppression of metabolism.

### Generating the predictive models

**Figure 3:**
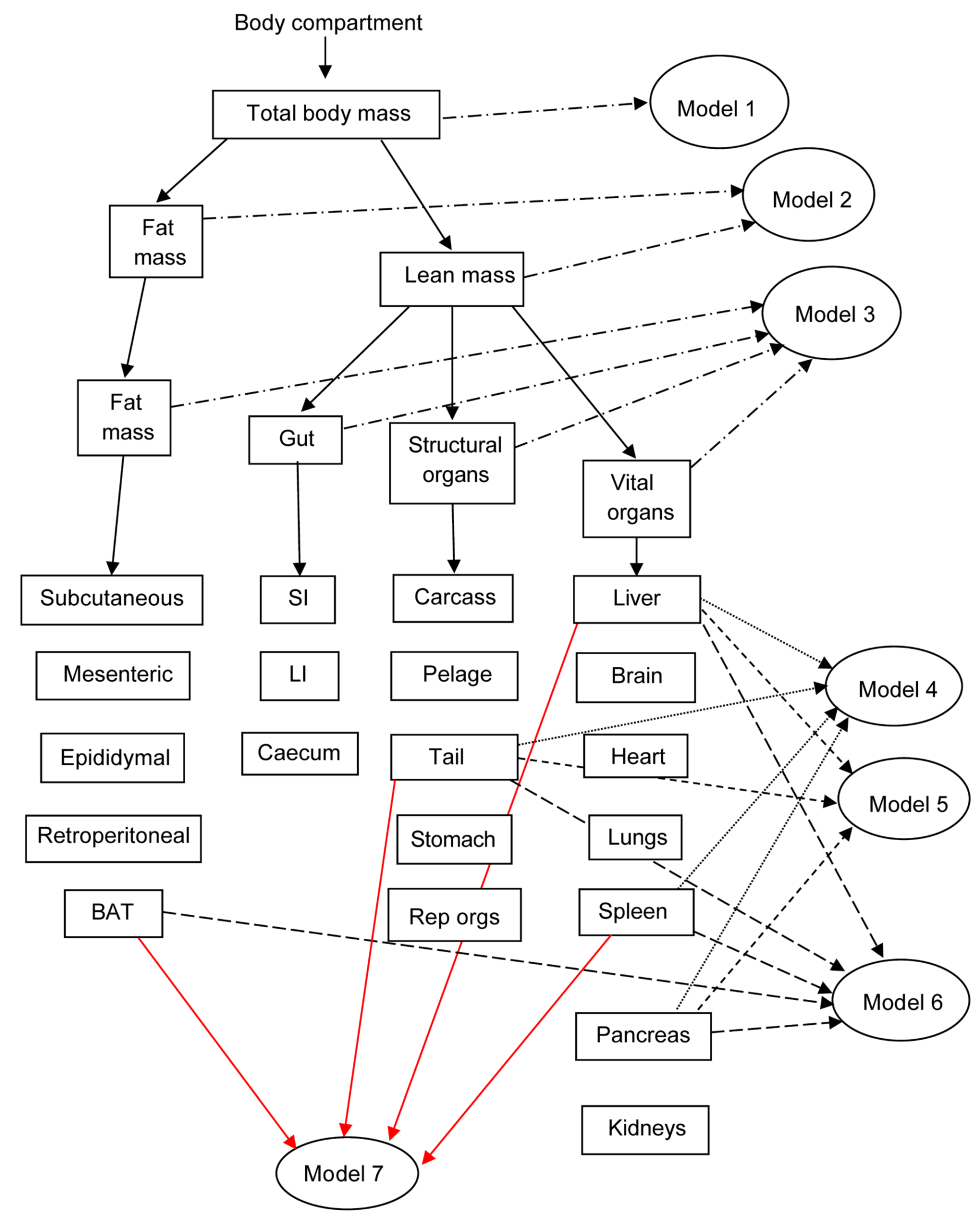
Diagram to show the inter-relationships of the different models based on body composition used to predict basal metabolic rate (BMR) in C57BL/6 mice Models 1 to 3 are based on gross weights of whole body (Model 1), separated into lean and fat mass (Model 2), or separated into 4 compartments (vital organs, structural tissue, fat tissue and the gut) (Model 3). Models 4 to 7 include various combinations of the different tissue weights in regression models that minimize the Akaike information criterion. SI and LI refer to small and large intestine respectively.

**Figure 4:**
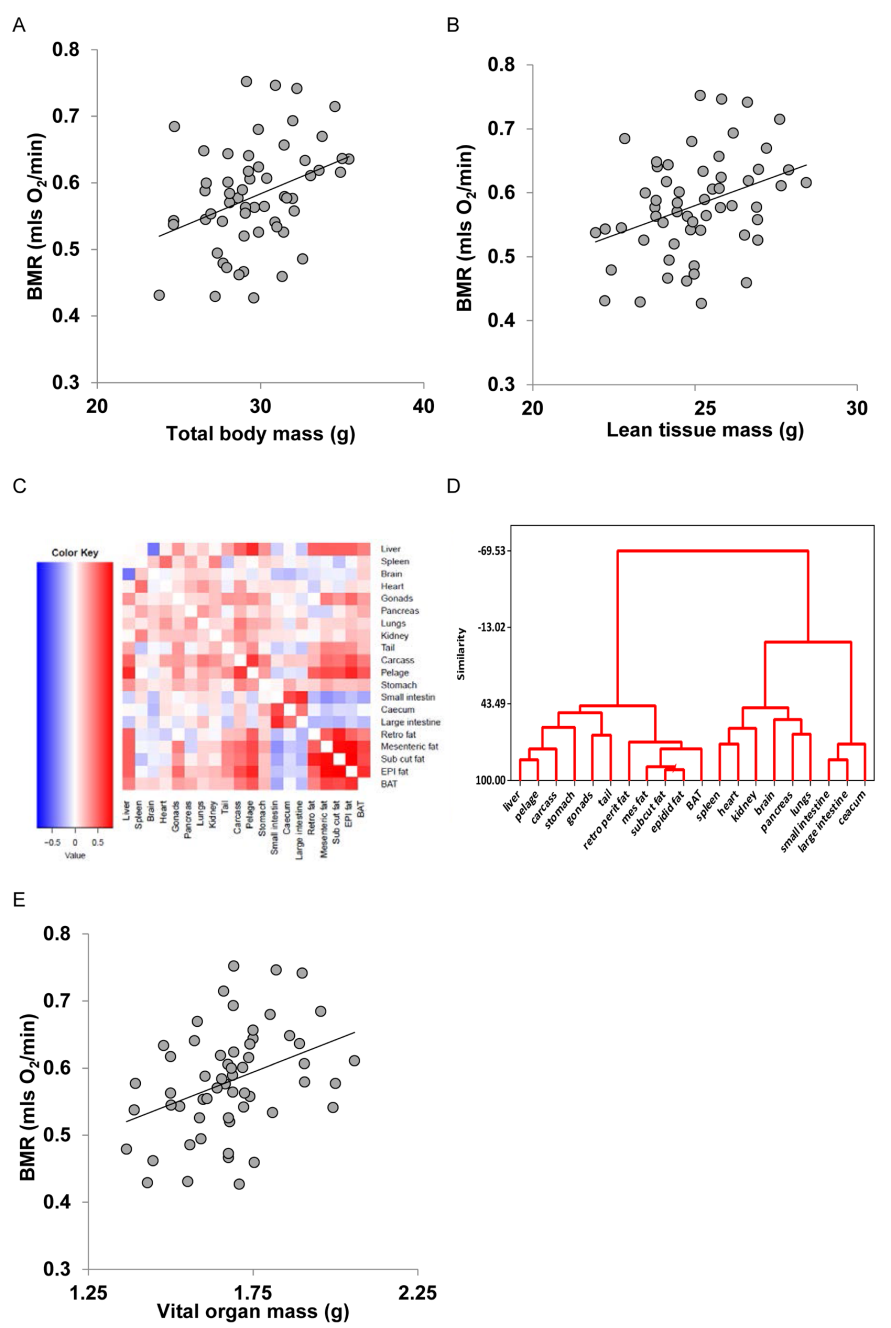
Basal metabolic rate (BMR) (mls O_2_/min) of 57 mice (strain C57BL/6) aged 5 months plotted against **A**. total body mass (g), **B**. lean body mass (g). **C**. correlation matrix between the masses of the different body components across 57 C57BL/6 mice used to construct models of basal metabolism. **D**. dendrogram derived from the correlation matrix in **C**., showing the groupings of the different tissues into 4 broad groups, and E: BMR (mls O_2_/min) plotted against mass of the summed vital organs (g). Fitted lines in figures **A**., **B**. and **E**. are least squares fit regressions. For details of statistics refer to text.

We generated seven models of BMR of increasing complexity based on the body composition (see methods) to explain the individual variation in BMR in a separate cohort of 57 C57BL/6 mice. The details of the models are illustrated in Figure 3. Model 1 included only total body mass (summed masses of all dissected components) of the mice as the predictor. There was a significant positive relationship (Figure 4A) between the BMR and the body mass, the least squares fit regression BMR = 0.2754 + 0.01028(body mass; g) explained 12.4% of the variation in BMR (F_1,55_ = 7.76, *P* = 0.007). Both the constant and the coefficient of the regression were significantly different from 0 (constant: *t* = 2.5, *P* = 0.015, coefficient: *t* = 2.79, *P* = 0.007). Model 2 included both lean mass and fat mass as predictors. In this case the fitted regression was BMR = 0.162 + 0.0159 (lean mass; g) + 0.00444(fat mass; g) which explained 13.3% of the variation in BMR (F_2,54_ = 4.14, *P* = 0.021). However the coefficient with respect to fat mass was not significant (*t* = 0.52, *P* = 0.604). The relationship between BMR and lean mass is shown in Figure 4B. To derive additional variables we performed a correlation and clustering analysis on the body composition data. The correlation matrix for these body compartments is shown in Figure 4C. From these data it is clear that the adipose tissue depots form a compartment that is strongly correlated with itself, but negatively related to the size of the alimentary tract components. Using this correlation matrix the clustering algorithm split the body components into 4 distinct groups (dendrogram in Figure 4D). These groups comprised the alimentary tract components (small and large intestine and caecum), adipose tissue stores (subcutaneous, epididymal, retroperitoneal and mesenteric white adipose tissue, and the brown adipose tissue (BAT)), vital organs (brain, kidneys, lungs, spleen, pancreas and heart) and mostly structural tissues (carcass, tail, pelage, reproductive organs, stomach and liver). To derive new predictors we used this classification. The new predictors were then the summed masses of the component tissues in each grouping. When used as independent predictors only the mass of the gut components was unrelated to the BMR (F_1,55_ = 1.16, *P* = 0.286). There were significant relationships to the summed fat mass (r^2^ = 0.073, F_1,55_ = 4.34, *P* = 0.042), summed structural tissue mass (r^2^ = 0.123, F_1,55_ = 7.70, *P* = 0.008) and the summed vital organ mass (r^2^ = 0.144, F_1,55_ = 9.24, *P* = 0.004: Figure 4E). However, when all 4 compartments were entered in a multiple regression analysis, only the vital organ and combined fat masses were significant predictors (F_2,54_ = 6.81, *P* = 0.002). Hence Model 3 was BMR = 0.2149 + 0.182(vital organ mass; g) + 0.01283(Fat mass; g).

**Figure 5:**
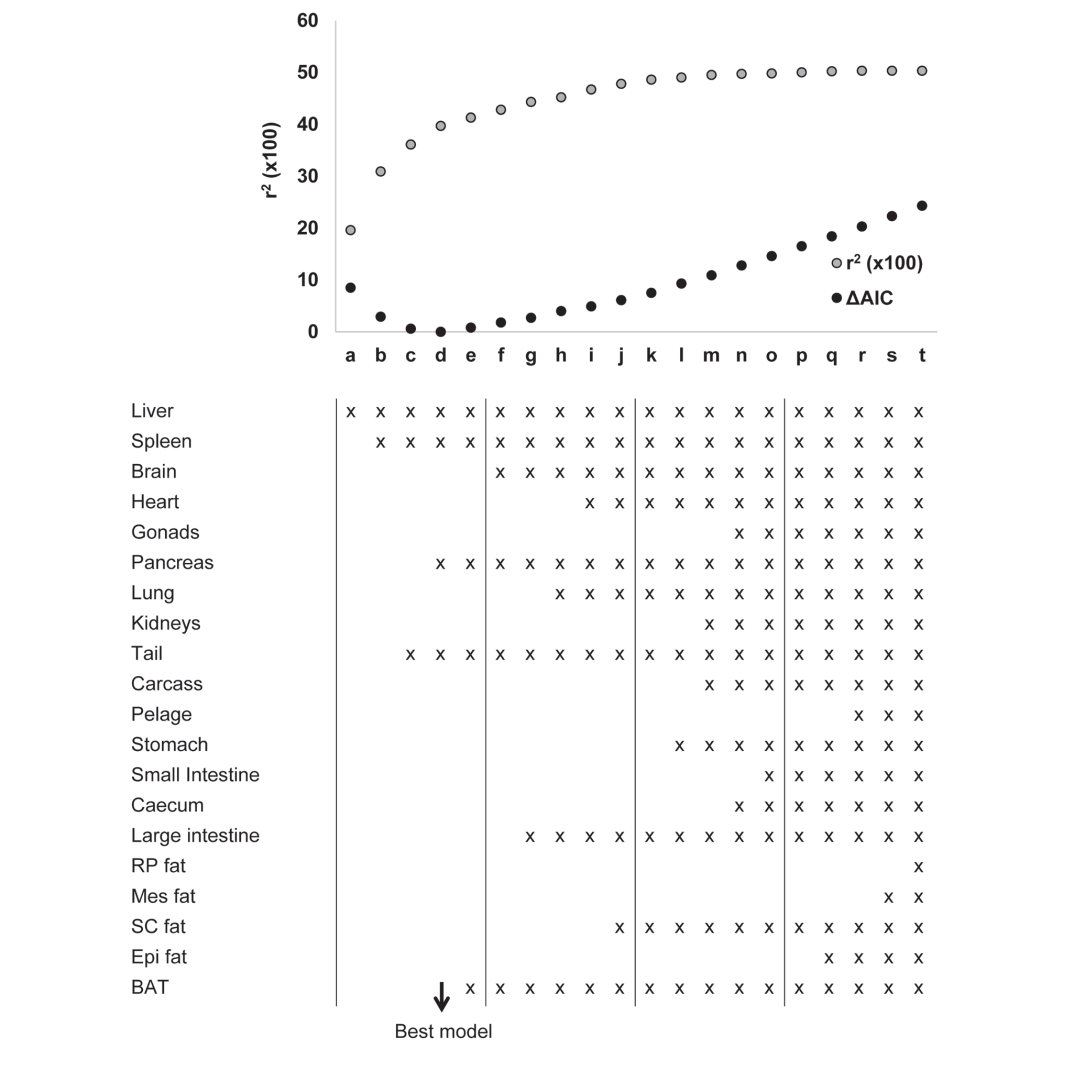
Modeling basal metabolic rate (BMR) from organ composition The plot shows the r^2^ (x100) (grey dots) and the delta Akaike information criterion (ΔAIC) (black dots) for each of 20 different models derived from stepwise regression performed by both backward elimination (models 4 to 20) and forward inclusion (models 1 to 4). The components of body composition included into each model are listed. Models 3, 4 and 5 minimized the ΔAIC and were used to predict metabolic rate of mice under calorie and protein restriction.

Models 4 to 7 were derived from different combinations of the individual organs. When the masses of individual organs were regressed against BMR, ten of them were significantly related to BMR (Table 1), although after correction for multiple testing only one remained significant (the liver). To derive predictions from the individual organ masses we used stepwise regression procedures to generate predictive equations systematically deleting terms from a full model including all 20 components, or systematically adding terms to a null model containing no predictors. Terms were added or deleted according to whether they contributed significantly to the explained variance or not. At each stage we calculated the explained variance in BMR (r^2^) and also the Akaike information criterion (AIC). We then calculated AIC relative to the lowest AIC value for the best fit model ( = ΔAIC). In total we evaluated 20 different combinations of predictors which we called models a to t. From these potential predictive models we selected four which became predictive models 4 to 7 for application to the CR and PR data. The relationships between r^2^, ΔAIC and the number of included predictors for these models (a) to (t) are shown in Figure 5. The best model (Model (d)) using the lowest ΔAIC as the criterion for model selection included the liver, spleen pancreas and the tail mass as predictors. The fitted regression equation was BMR = −0.060 + 0.11952 (liver mass; g) + 1.6206(spleen mass; g) +0.1990(pancreas; g) + 0.3826(tail; g), and this explained 39.7% of the variation in BMR (F_4,52_ = 8.57, *P* < 0.0005). We selected this model for predicting the BMR of the CR mice and in that context called it Model 4. Two other groupings of predictors had ΔAIC values less than 1. These were Model (c) (ΔAIC = 0.6) which included liver, spleen and tail masses, and Model (e) (ΔAIC = 0.8) which included liver, spleen, pancreas tail and BAT masses. The respective least squares fit regression equations were for Model (c): BMR = −0.0254 + 0.12663(liver mass; g) + 1.833 (spleen mass; g) + 0.3793(tail mass; g) which explained 36.1% of the variation in BMR (F_3,53_ = 9.98, *P* < 0.0005). For Model (e), the equation was BMR = 0.0362 + 0.09547(liver mass; g) + 1.5946 (spleen mass; g) + 0.3627(tail mass; g) + 0.1681(pancreas mass; g) + 0.1736 (BAT mass; g) which explained 41.3% of the variation in BMR (F_5,51_ = 7.19, *P* < 0.0005). We selected these two models also to predict the BMR of the CR and PR mice and renamed them respectively in that context Models 5 and 6. We then used ‘best subsets’ regression to explore whether any other combinations of predictors had a ΔAIC < 1.0 and this identified one additional grouping (liver, spleen, tail and BAT) with ΔAIC = 0.6). The relevant regression equation was BMR = −0.0017 + 0.0946(liver mass; g) + 1.7593 (spleen mass; g) + 0.3546(tail mass; g) + 0.2211 (BAT mass; g) which explained 38.9% of the variation in BMR (F_4,52_ = 8.26, *P* < 0.0005). We also selected this model for prediction of BMR in the CR and PR mice and in that context named it Model 7. Hence from the independent sample of mice we ended up with seven different predictive models. The inter-relationships of these models are illustrated in Figure S1 in the Supplementary Materials.

**Table 1:** Correlation analysis of the relationships between individual organ masses and BMR in C57BL/6 mice

Organ	r	*p*	sig	adj sig
Liver	0.443	<0.001	***	*
Spleen	0.378	0.004	**	
Brain	−0.018	0.893		
Heart	0.163	0.226		
Gonads	0.244	0.067		
Pancreas	0.307	0.02	*	
Lungs	0.202	0.132		
Kidneys	0.301	0.023	*	
Tail	0.278	0.036	*	
Carcass	0.232	0.083		
Pelage	0.342	0.009	**	
Stomach	0.303	0.022	*	
Small Intestine	−0.133	0.324		
Large Intestine	−0.187	0.164		
Caecum	−0.022	0.87		
Retro WAT	0.195	0.145		
Mes WAT	0.262	0.049	*	
SC WAT	0.208	0.12		
Epi WAT	0.303	0.022	*	
BAT	0.391	0.003	**	

Significance (sig) denotes the p value in the following ranges *** < 0.001, ** < 0.1 >0.001, * < 0.05 > 0.01. adj sig refers to the Bonferroni corrected significance level. WAT is white and BAT Brown adipose tissue. Retro is retroperitoneal, Mes is mesenteric, SC is sub-cutaneous and Epi is epididymal.

### Comparing measured BMR in CR and PR mice to predicted BMR from the predictive models

We used the 7 models (Figure 3) derived from the analyses of the independent set of mice to derive predicted BMRs from the body composition data of the mice under CR and PR. We then compared the predicted and observed BMRs and explored whether the differences between prediction and observation reflected suppression or enhancement of BMR, whether this difference was systematically linked to the level of CR or PR, and whether it was also associated with circulating hormone levels and body temperature. A summary of the results for all 7 models is provided in Table 2.

**Table 2:** Summary of the comparison between the observed BMR for mice under CR and the predicted BMR using the seven different predictive models

Model	r^2^	Intercept	p(int)	Gradient	p(grad)
ONE	0.667	−0.697	*p* < 0.0005	2.294	*p* < 0.0005
TWO	0.667	−0.433	*p* < 0.0005	1.800	*p* < 0.0005
THREE	0.713	−0.667	*p* < 0.0005	2.431	*p* < 0.0005
FOUR	0.537	−0.035	*p* = 0.559	0.939	*p* = 0.77
FIVE	0.528	0.0367	*p* = 0.567	0.887	*p* = 0.38
SIX	0.554	0.0159	*p* = 0.800	0.927	*p* = 0.45
SEVEN	0.557	−0.051	*p* = 0.470	0.963	*p* = 0.84

R^2^ is the correlation coefficient squared for the least squares fit regression, intercept is the intercept of the regression, p(int) is the probability that the intercept differs from 0 (t-test), Gradient is the gradient of the regression and p(grad) is the probability that the gradient differs from 1.0 (t-test). Parameters lists the parameters included in the predictive equations for the respective models. For details of the different predictive models refer to the methods and Figure S1 in Supplementary Materials.

#### Model 1 (body mass)

**Figure 6:**
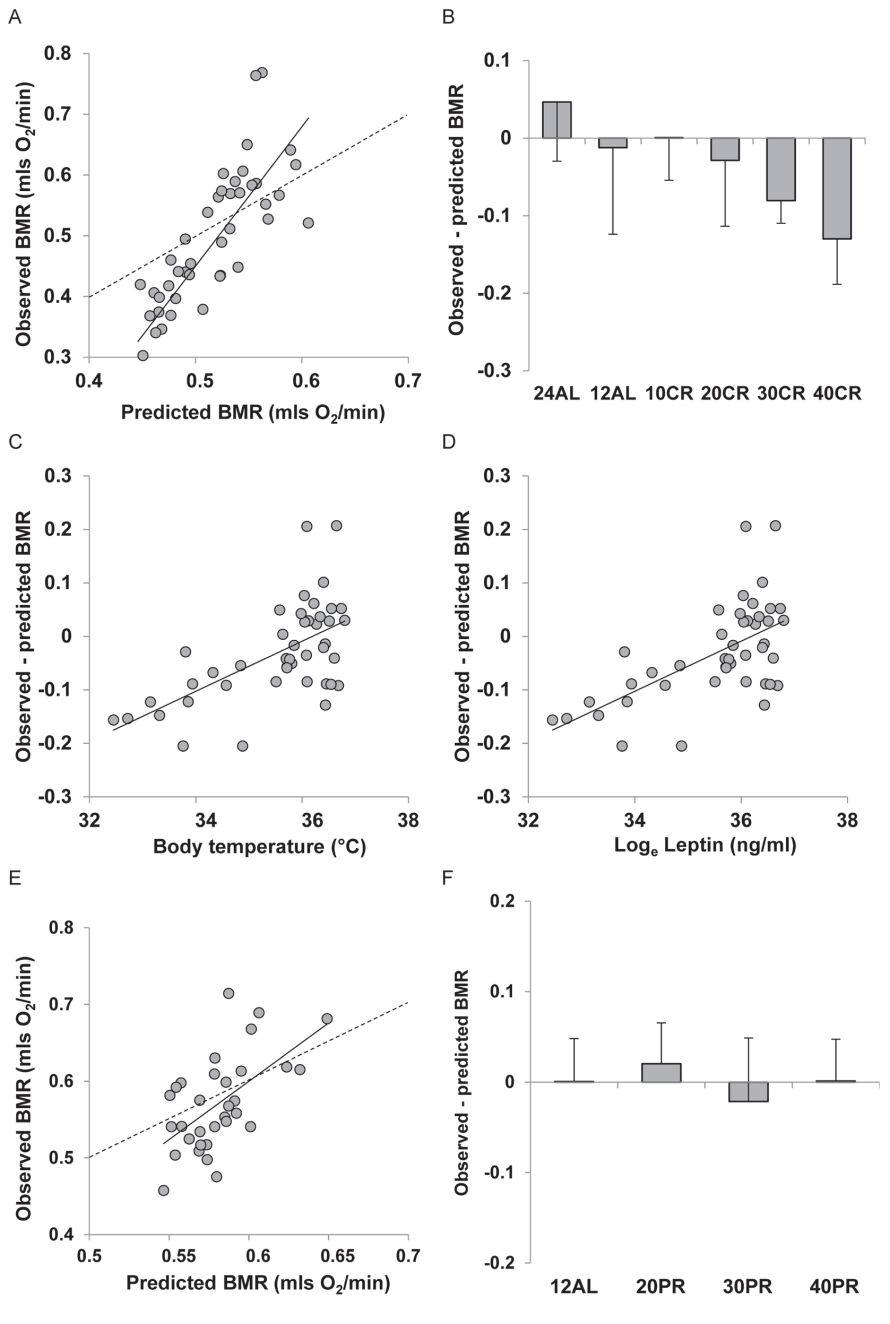
Comparison of observed basal metabolism after calorie or protein restriction (CR or PR) and the predictions of Model 1 based on total body mass **A**. basal metabolic rate (BMR) (mls O_2_/min) observed after 3 months of CR and **E**. 3 months of PR plotted against the prediction using Model 1. Dashed line is line of equality and solid line is least squares fit regression (for details see text). Deviations of observed metabolic rate from the model prediction in relation to **B**. the level of CR and **F**. the level of PR. Relationships between the difference between the observed metabolism and that predicted from the model and **C**. body temperature (^°^C) and **D**. log_e_ circulating leptin levels (ng/ml).

There was a strong positive relationship between the predicted BMR from Model 1 and the measured BMR of the mice that had been under CR (Figure 6A). The least squares fit regression: Measured BMR = −0.6973 + 2.294(Model 1 Predicted BMR), explained 66.7% of the variation in the measured BMR (F_1,45_ = 89.12, *P* < 0.0005). The coefficient of the fitted relationship was significantly > 1 (*t* = 5.32, *P* < 0.0005) and the intercept was significantly different to 0 (*t* = −5.59, *P* < 0.0005). The differences between the Model 1 predictions and the observed BMRs were strongly related to the CR treatment group (ANOVA F_5,41_ = 5.79, *P* < 0.0005) with a progressive discrepancy as the level of restriction increased (Figure 6B) indicating increasing suppression of BMR. In addition the difference between the prediction and the observed metabolism was positively correlated to the body temperature averaged over the last 20 days of restriction (r^2^ = 0.371, F1,42 = 24.77, *P* < 0.0005: Figure 6C) and was also positively related to the levels of circulating leptin (*t* = 3.76, *P* < 0.001: Figure 6D) and negatively to circulating resistin (*t* = −2.1, *P* = 0.042) but was not significantly associated with circulating levels of any other measured hormone including insulin, tumor necrosis factor (TNF)-α, interleukin (IL)-6, and insulin-like growth factor (IGF-1). For the mice under PR there was also a strong relationship between the prediction from Model 1 and the observed BMR after 3 months of PR. The least squares fit regression Measured BMR = −0.3007 + 1.4242(Model 1 Predicted BMR) explained 33.9% of the variation in the measured BMR (F_1,30_ = 15.39, *P* < 0.0005: Figure 6E). The difference between the prediction and the observed BMR was not significantly related to the level of PR (ANOVA: F_3,28_ = 0.49, *P* = 0.694: Figure 5F).

#### Model 2 (lean and fat mass)

**Figure 7:**
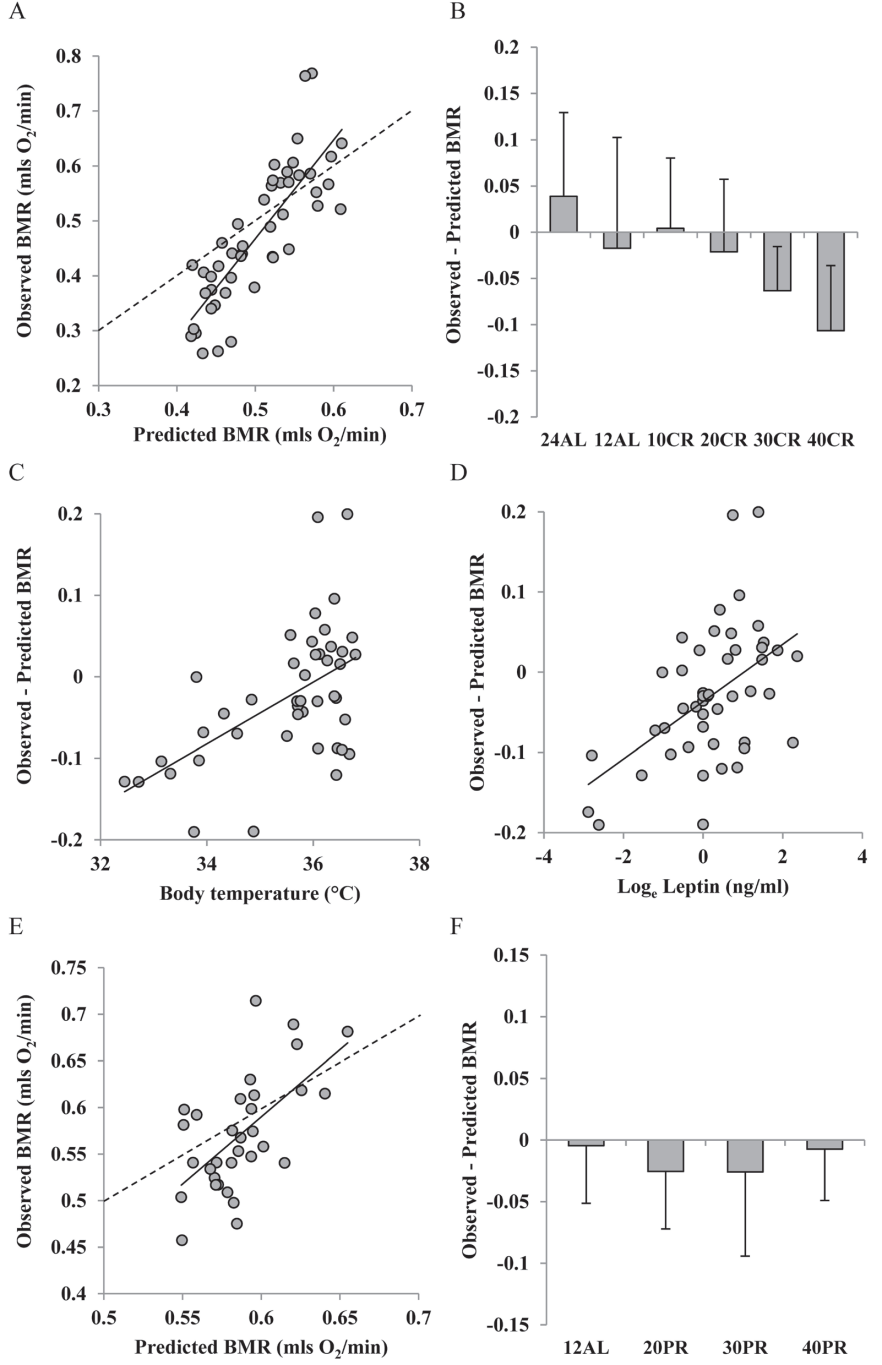
Comparison of observed basal metabolism after calorie and protein restriction (CR and PR) and the predictions of Model 2 based on lean and fat mass **A**. and **E**. basal metabolic rate (BMR) (mls O_2_/min) observed after A, 3 months of CR and **E**, 3 months of PR plotted against the prediction using Model 2. Dashed line is line of equality and solid line is least squares fit regression (for details see text). Deviations of observed metabolic rate from the model prediction in relation to **B**., the level of CR and **F**: the level of PR. Relationships between the difference between the observed metabolism and that predicted from the model and **C**. body temperature (^°^C) and **D**. log_e_ circulating leptin levels (ng/ml).

There was a strong positive relationship between the predicted BMR from Model 2 and the measured BMR of the mice that had been under CR (Figure 7A). The least squares fit regression: Measured BMR = −0.433 + 1.8009(Model 2 Predicted BMR), explained 67.7% of the variation in the measured BMR (F_1,45_ = 94.23, *P* < 0.0005). The coefficient of the fitted relationship was significantly > 1 (*t* = 9.71, *P* < 0.0005) and the intercept was significantly different to 0 (*t* = −4.6, *P* < 0.0005). The differences between the Model 2 predictions and the observed BMRs were strongly related to the CR treatment group (ANOVA F_5,41_ = 4.09, *P* < 0.004) with a progressive discrepancy as the level of restriction increased (Figure 7B) indicating increasing suppression of BMR. In addition the difference between the prediction and the observed metabolism was positively correlated to the body temperature averaged over the last 20 days of restriction (r^2^ = 0.288, F_1,42_ = 17.02, *P* < 0.0005: Figure 7C) and was also positively related to the levels of circulating leptin (*t* = 3.22, *P* < 0.003: Figure 7D) but was not significantly associated with circulating levels of any other measured hormone including insulin, TNF-α, IL-6, resistin, and IGF-1. For the mice under PR there was also a strong relationship between the prediction from Model 2 and the observed BMR after 3 months of PR. The least squares fit regression Measured BMR = −0.2575 + 1.3051(Model 2 Predicted BMR) explained 36.4% of the variation in the measured BMR (F_1,30_ = 17.2, *P* < 0.0005: Figure 7E). The difference between the prediction and the observed BMR was not significantly related to the level of PR (ANOVA: F_3,28_ = 0.45, *P* = 0.718: Figure 7F). The response of Model 3 (clustered tissues) was similar to that of Model 2. Details are included in Supplementary Materials and Figure S1.

#### Model 4 (best fit lowest AIC criterion model including liver, spleen, pancreas and tail as predictors)

**Figure 8:**
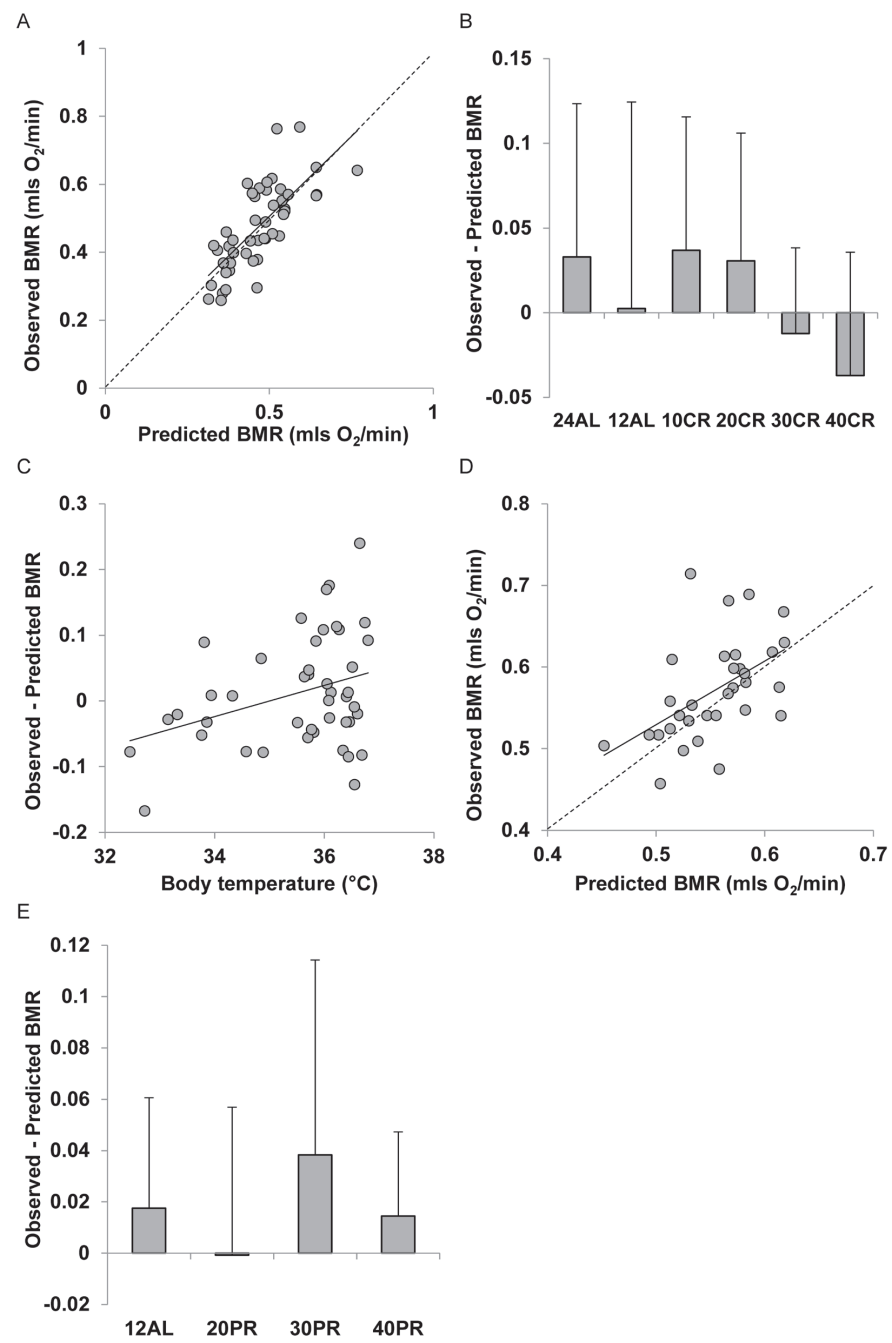
Comparison of observed basal metabolism after calorie or protein restriction (CR and PR) and the predictions of Model 4 based on the body composition prediction model with the lowest AIC score (using masses of liver, spleen, tail and pancreas) **A**. and **D**. basal metabolic rate (mls O_2_/min) observed after **A**. 3 months of CR and **D**. 3 months of PR plotted against the prediction using Model 4. Dashed line is line of equality and solid line is least squares fit regression (for details see text). Deviations of observed metabolic rate from the model prediction in relation to **B**., the level of CR and **E**. the level of PR. **C**. Relationship between the difference between the observed metabolism and that predicted from the model body temperature (^°^C).

Measured BMR was also strongly positively related to the predicted BMR from Model 4 for the mice that had been under CR (Figure 8A). The least squares fit regression Measured BMR = −0.0365 + 0.939 Model 4 Predicted BMR explained 53.7% of the variation in the measured BMR (F_1,45_ = 52.25, *P* < 0.0005). The intercept of this relationship was not significantly different from 0 (*t* = 0.59, *P* = 0.559) and the coefficient was not significantly different from 1 (*t* = 0.469, *P* = 0.77). The differences between the Model 4 predictions and the observed BMRs were not significantly related to the CR treatment group (ANOVA F_5,41_ = 1.01, *P* = 0.425, Figure 8B). Nevertheless, despite this lack of a group effect the discrepancy between the Model 4 prediction and the observed BMR was positively related to the body temperature averaged over the last 20 days of restriction (r^2^ = 0.109, F_1,42_ = 5.14, *P* = 0.029: Figure 8C). However, the differences were not significantly related to any of the measured circulating hormones. For the mice under PR there was also a significant relationship between the prediction from Model 4 and the observed BMR after 3 months of PR. The least squares fit regression Measured BMR = 0.1394 + 0.780 Model 4 Predicted BMR explained 26.2% of the variation in the measured BMR (F_1,30_ = 10.67, *P* = 0.003: Figure 8D). The difference between the prediction and the observed BMR was not significantly related to the level of PR (ANOVA: F_3,28_ = 0.69, *P* = 0.566: Figure 8E). Models 5, 6 and 7 showed similar patterns to Model 4. Full details are available for these models are available in Supplementary Materials and Figures S2 to S4.

## DISCUSSION

During CR there is an energy deficit, and animals need to lower their energy demands to bring expenditure and intake back into balance. As has been observed many times previously one way that animals achieve this is to lower their BMR [13, 15, 25] and reviewed in [45]. Consistent with these earlier data the raw unadjusted BMR values showed a profound decline in relation to the level of CR. We did not observe the same trend in the mice that had been exposed to PR. We can therefore infer that the reduced BMR in mice that experienced CR was due to the energy deficit they encountered and was not a response to the simultaneously lowered levels of protein in their diets. It is also generally observed that mice under CR also lose body weight and change their body composition [46, 47] and in this respect the mice studied here were no different [35]. The question arises therefore as to whether the decline in BMR under CR is a consequence only of the altered body mass and tissue composition, or whether there is additionally some suppression of metabolism also occurring at the tissue level [48], and if so what drives this metabolic suppression?

We used three classical ratio methods to normalize the BMR measures - these included dividing the metabolism by Mass^0.75^, Mass^0.66^ and dividing by the lean tissue mass. The pattern that was revealed in all three cases was the same (Figure 2). Even when the BMR was ‘normalized’ for the weight change there was still a reduction in the BMR in relation to the extent of restriction. Using the same calculations for the mice under PR revealed no such suppression. These data would conventionally be interpreted as indicating there was some progressive suppression of the metabolism happening at greater levels of CR, that was absent under PR. Since higher levels of CR in rodents are positively linked to greater lifespan [1, 23, 49, 50] one interpretation that could be drawn is that the suppression of BMR is possibly causally associated with the extended lifespan. This would be the classical ‘rate of living’ type interpretation of the impact of CR. Since the levels of PR that occur simultaneously under CR do not generate a significant lifespan effect [50] the absence of a suppression effect on the PR animals would be consistent with this interpretation.

Normalizing BMR by using ratio methods has a well-established set of problems that may result in spurious interpretations of the changes in metabolic rates [33, 40, 51]. We therefore used a different approach to address this issue. This involved creating a set of predictive equations that linked together aspects of the body composition of a separate set of C57BL/6 mice with their BMR. We then used these equations to predict the metabolic rates under CR and PR from the known body compositions of these animals [35] and compared them to the measured BMR. The derived models had many features consistent with previous studies where BMR has been linked to body composition. First, when body mass alone was used as a predictor there was a non-zero intercept for the fitted regression [52, 53]. Second, when lean and fat mass were used as predictors, lean mass variation explained much more of the variance in BMR than the fat mass [39, 54, 55]. Finally the pooled vital organ masses were a much better predictor than the lean mass reflecting their much greater tissue specific rates of metabolism [56, 57]. Using the simple models that were based on gross features of the body composition (ie Model 1: body mass (Figure 5), Model 2 lean and fat mass (Figure 6), and Model 3 vital organ mass (Figure 7)) the patterns were all similar. Although the predictions explained an impressive amount of the variation in the observed BMR (63 to 67%) the relationships all had gradients that exceeded 1, and intercepts that were significantly different from zero. The discrepancies between the prediction and the observations were systematically associated with the extent of the CR (but not PR). These trends also pointed to progressive suppression of the metabolism in the mice under CR - consistent with the findings when using the ratio approaches. Moreover, there was a significant relationship in all 3 cases between the discrepancy in the metabolism and the body temperature [31] and between the discrepancy and circulating leptin levels [41]. These models then make a cogent story that under CR there is a progressive reduction in fat mass, which leads to a reduction in leptin levels which drives a suppression of BMR [39] that exceeds the expected reductions based on changes in body composition alone. This suppression of metabolic rate then results in lowered body temperature and this may be causally linked to the observed lifespan effect [58]. These relationships have been inferred previously for rodents [48, 59, 60], non-human primates [25, 61, 62] and humans [63–66] when on CR protocols. On the other hand, the absence of any fat loss in the PR animals [35] does not lead to a reduction in leptin levels [41] resulting in no suppression of metabolic rate (confirmed here) and hence no lowering of body temperature [31] consistent with the absence of a lifespan effect at these levels of PR [50].

It is a nice and internally consistent story that matches aspects of the wider literature [25, 47, 48, 60–66] but our further analysis using more detailed body composition models suggests that it is wrong. When we explored the relationships between individual organ masses and the BMR our data showed some patterns consistent with previous such models. In particular the liver turned out to be a major predictor of the metabolism (as also observed by [37, 40]. In addition the tail also turned out to be an important predictor. It is worth noting that in small rodents the tail is a thermoregulatory organ involved in heat dissipation. Consistent with the numerous suggestions that BAT is a major contributor to the level of BMR [67, 68] we found that BAT mass entered 2 of the 4 best predictive models (based on the models with lowest AIC). It is interesting that these features are all related to heat production during thermoregulation. Finally, when we included such predictors the non-zero intercept of the regression Model disappeared, consistent with the suggestion that the non-zero intercept is a consequence of the complex changes in body composition as animals change their body size [53].

When we used these 4 models that were based on the detailed body composition to predict the observed BMR we explained a similar amount of the variation in the observed BMR as we did with the less complex models (55 to 67%). However, in these cases the slopes of the relationships between prediction and observation were much closer to 1 (not significantly different), and the intercepts much closer, and not significantly different, to 0. The consequence was that with these models the discrepancy between prediction and observation was in all 4 cases unrelated to the level of restriction. Moreover while there was a weak link between the discrepancy and body temperature for 2 of the 4 models (not significant if adjusted for the 4 performed tests) in none of the cases was the discrepancy associated with the measured levels of circulating hormones in the same individuals. The implication is very clear. The apparent suppression of metabolism linked to leptin levels and body temperature was an artifact of using inadequate models to correct for the body composition changes. When more detailed body composition models were used then the apparent ‘suppression’ of BMR in relation to the level of restriction disappeared. Hence we conclude that although the absolute level of BMR declines when mice are under CR (but not under PR) this reduction can be completely explained by the detailed changes in body composition as the mice lose weight. There was no evidence of any extra tissue level suppression of metabolism, and hence this suppression cannot be invoked as a potential causal factor linked to the increased longevity and healthspans of mice under CR. These data contrast the work of Selman *et al* (2005) who suggested that metabolic rates of rats under CR were actually higher than predicted from a model based on body composition change [40]. The principal difference between these studies was that we studied BMR while Selman *et al* (2005) studied total daily energy expenditure which includes energy demands due to physical activity. These patterns do not necessarily need to accord with each other. In fact BMRs measured here comprised on average only 33.2% of the total energy expenditure at baseline, leaving a large scope for metabolic adaptation to occur in other components of the energy budget.

Our observations have wide implications well beyond the study of CR and aging. Many studies have been performed where a gene has been knocked out, or transgenically over expressed, and some impact has been inferred on the metabolic rate (often by using a ratio method but more recently increasingly by using ANCOVA based methods: [25, 33]. What the current study shows is that in all these cases the impact on basal metabolism might in fact be traced to an effect on the detailed body composition of the animals in question, with no actual tissue level impact on basal (or resting) metabolic rate. We are aware of no studies where an approach like that used in the current paper has been used to eliminate this as a possibility. Until more such studies are performed it will be unclear which previous studies that have inferred impacts on tissue level energy expenditure are safe, and which are not. An additional complementary approach to explore this issue may be to use different techniques to get at the tissue level metabolism, such as uptake of radiolabeled tracers in the live animal or metabolic rates of tissues *ex vivo*.

## MATERIALS AND METHODS

### Animals

All procedures were reviewed and approved by University of Aberdeen ethical approval committee and carried out under a Home Office issued license compliant with the Animals (Scientific Procedures) Act 1986. Experiments and this report were conducted consistent with the ARRIVE guidelines. The rationale and design of the overall graded restriction study has been detailed previously [35]. In brief male 48 C57BL/6J mice (Charles River, Ormiston, UK) were acclimated for 6 weeks prior to implantation of transmitters at 12 weeks of age, after which we allowed 4 weeks recovery time prior to experimentation. A number of baseline measurements, including dual X-ray absorptiometry (DXA) for body composition, glucose tolerance tests (GTT) and BMR, were carried out at 17-18 weeks old. Over the baseline period all animals were provided with *ad libitum* access to water and open source diet (D12450B, Research Diets, NJ, USA) containing 20% protein, 70% carbohydrate and 10% fat (by energy).

CR or PR was started at 20 weeks of age, approximately equivalent to early adulthood of humans. In the CR study all mice continued to be fed D12450B with restriction levels set at 10%, 20%, 30% and 40% (referred to as 10CR, 20CR, 30CR and 40CR) of individual baseline food intake. Final numbers for the groups were *n* = 8 for the 10CR and 20CR groups, *n* = 7 for the 30CR (one mouse died) and *n* = 9 for the 40CR. The rationale for using graded levels of restriction was that in rodents increasing levels of restriction lead to increases in lifespan in a linear fashion [23, 50] hence traits that vary in linear relation to the level of restriction are potential mediators of the lifespan effect. The aim of the graded CR study was to construct a multi-level description of the response to graded levels of caloric restriction. Clearly there is no procedure to perform a power analysis for a ‘multi-level descriptive analysis’. Sample size for each level of the graded restriction was therefore based on a power analysis for a single responsive trait (change in body fat content) under the assumption that alpha = 0.05, Power = 0.8 to detect a 30% difference in fat contents with a projected analysis using one-way ANOVA, and the prior estimated within group CV of 15%. This suggested a sample size of 8 per group. For the PR study diets were designed to match the reduced protein level of the 20CR, 30CR and 40CR, i.e. protein content was equivalent to 16, 14 and 12% protein (made up by increased carbohydrate) (D13020201, D13020202 and D13020203 respectively, Research Diets, NJ, USA). These are referred to as 20PR, 30PR and 40PR. Mice may compensate for the reduced protein intake by overeating [69, 70]; this was prevented by feeding a fixed weight of food equivalent to their own individual baseline intake on D12450B (20% protein). All four diets were isocaloric (3.8kcal/g) and the duration of PR phase was also 3 months. The rationale for also studying PR alongside CR is that some studies have suggested that the effect of CR is because of the concomitant restriction of protein intake [69, 70]: but see [50]. Comparing the responses of mice to both CR and PR allows us to distinguish which responses are common to CR and PR and which are unique to the individual treatments.

For the CR study we used two control groups that had *ad libitum* food access. For one group (24AL) the food was available 24h per day. For the second group food was available *ad libitum* but only during the 12 hours of darkness (12AL). *n* = 8 for both the control AL groups. This procedure is also called time restricted feeding in some studies [72]. Apart from the 24AL group, mice were fed once per day, immediately prior to lights out and food was removed at the onset of light phase. Because mice under CR generally consume all their food within a few hours of it being provided [73, 74], but animals with 24h access to food eat about 20% of their food during daylight (unpublished data) there is a potential confound between the effect of long term restriction and the effect of short term starvation when comparisons are made between animals under CR and those fed completely ad libitum (ie 24AL). Since 12AL mice had no food from the start of the light phase, this allowed us to control for short term starvation effects in assays performed in the mid-afternoon. Both CR and PR studies utilized a control 12AL group with animals treated identically. Several parameters were made at the end of the restriction period (last week of restriction) including BMR which is the focus of the current paper. Mice were then killed on day 90 of restriction, urine and blood samples were collected, a complete body dissection into 20 separate components was made, and tissues were stored at −80 ^°^C for further analyzes. In the dissection data we located 22 outliers. This was 1.37% of the total measurements (20 organs x 80 individuals). These were almost all morphologically impossible miss keyed data e.g. 1.33g for weight of the brain instead of 0133g and were corrected by cross checking to the original notebooks where the data were reported. A major strength of the overall study is that we have performed multiple assays in the same individuals. Previous studies of these individual mice have concerned body composition [35], endocrine and oxidative stress related parameters [41], body temperature [31], behavior patterns [42], physical activity [14]and global transcriptomic patterns in the hypothalamus [43, 44]. Animals were randomized into treatment groups. It is not possible to blind individuals who feed the mice from the nature of the treatments. However, once the animals were sacrificed and tissues collected all tissue samples and other data were linked to the individual mouse by an ID number which did not reveal their group membership, and hence such assays were conducted blind of the treatment.

In a completely independent group of 60 C57BL/6 mice aged 8 months we measured BMR and the mice were then culled and dissected using the same protocol for the mice under CR and PR [35]. The aim of this separate cohort of mice was to construct predictive models of BMR from the body composition. The sample size of 60 was based on the assumption that most models would have 1 to 6 predictors and a rule of thumb for multiple regression is that ideally there should be at least a tenfold greater number of observations than predictors. Data for 3 animals were rejected because they did not settle down in the respirometry chamber and hence did not provide a valid estimate of BMR. Hence the final sample was 57. For the dissection data we identified 13 outliers. This was 1.14% of values (20 organs x 57 individuals). These were almost all miss keyed data and were again corrected by cross checking to the original notebooks where the original data were recorded. We could then apply these predictive models to the mice under CR and PR using the detailed body composition to predict the expected level of BMR and then compare this prediction to the observation to establish if any metabolic suppression had occurred (see also [40] for the same approach applied with respect to daily energy demands in rats). Model construction is detailed under statistical analysis below.

### Basal metabolic rate

BMR is the post absorptive metabolism of a non-torpid animal at rest within the thermoneutral zone [75]. Mice were food deprived for at least 5 but not more than 12 hours prior to the measurements. This is sufficient for mice to be post absorptive, but not so long that they enable anti-starvation measures to suppress metabolism [76] There was no impact of variation in this duration on the resultant BMR measurements [15]. Oxygen consumption and CO_2_ production were measured using small custom built flow through respirometer chambers. Each respiration chamber was a Perspex cylinder with a volume of 1L, attached to a dedicated Servomex gas analyzer measuring both O_2_ and CO_2_ levels (Servomex 1100A or Xentra 4100; Servomex Ltd, UK) Each chamber was ventilated with a metered flow rate between 450 and 600 mls/minute (Mass-flow controllers (MKS Instruments, Cheshire, UK)) with fresh air from outside the building that was dried using silica gel, and measured using a calibrated Alexander Wrights Ltd precision test meter (DM3A, accurate to 0.05%). Tubing volumes between the chamber and analyzer were negligible. The analyzers were calibrated with oxygen free nitrogen, 5% CO_2_ in nitrogen (BOC special gases) and outside air (20.95% oxygen) prior to every measurement. Gases were dried prior to being measured but we did not scrub the CO_2_ (as recommended by [77]). Because each chamber had a dedicated analyzer there was no downtime due to switching flows from multiple chambers to the analyze, plus the flow rate to volume ratio meant that the response time of the system was very fast, allowing us to easily diagnose between periods when the animals were active and when they were at rest (independently validated in the same system against movement records from implanted transmitters by [78]). The lower critical temperature of mice depends on their body mass [79]. Since we had a range of mice varying from around 17 to 35g we used a temperature of 30 ^°^C for all the measurements, meaning all mice were within their thermoneutral zone as required for BMR measures, although their position within the zone was probably dependent on their body mass. Chambers were housed within an incubator (Gallenkamp, Loughborough, UK). Mice under 30 and 40% CR often fall torpid during the daytime 31]. Torpor in a mouse in a fast response analyzer of the type we used is easily diagnosed by a characteristic fall in O_2_ and CO_2_ levels almost to baseline, and we inspected all traces to ensure that mice were not torpid during the measurement periods. Measurements were made at 30 s intervals for 180 min. The ‘lowest’ metabolic rate is a function of how wide the averaging window that is used for the measurements [80]. We averaged the measurements over a 10 measurement window (5 minutes of metabolism) and located the absolute lowest metabolic rate and also the metabolic rate with the lowest variation over the 10 measurements. We did this because some mice occasionally show transient dips in metabolism [78] and these give a spurious indication of the lowest metabolic rate. Normally these two measures coincided closely but when they didn't we used the measure with lowest variation. Measurements using this system show high repeatability in repeated measures of individuals [78]. Lowest oxygen consumption was strongly correlated with the lowest CO_2_ production and hence also the lowest inferred energy expenditure, we therefore present here only the analysis for oxygen consumption (mls O_2_/minute) and have called this BMR throughout the paper. Analyses based on CO_2_ production or inferred energy expenditure yields almost identical results.

### Statistical analysis: model construction and evaluation

Using the data for the separate series of mice that were not involved in CR or PR experiments we built a series of predictive models of increasing complexity. The simplest model (Model 1) involved using the total body mass (total weight of all dissected organs) as a predictor in a simple least squares regression model. The second model (Model 2) included 2 predictors (lean tissue mass and fat tissue mass) in a multiple regression analysis. We then produced a correlation matrix for all the body components resulting from the dissection, and entered these data into a clustering analysis. We used the correlation matrix and the ‘Ward’ linkage method, which minimizes the within cluster sum of squares, to generate the dendrogram. We also used ‘average’ and median linkage methods and the clustering of variables was not sensitive to the linkage method. This identified 4 different clusters of variables which functionally appeared to represent structural tissues, adipose tissues, vital organs and the alimentary tract. The only organs that appeared to be misclassified by this procedure with respect to their biological functions were the liver and stomach, which were both included by the analysis into the ‘structural’ cluster. We calculated the summed weights of the organs within each cluster to generate 4 new variables: named structural, fat, vital organs and gut. For Model 3 we then performed a multiple regression analysis with the weights of these four ‘new’ variables as the predictor traits.

The remaining 4 models (Models 4 to 7) were derived using different combinations of the individual organ weights as predictors. To include the individual organ weights as predictors we proceeded by performing stepwise regression analysis using forward inclusion (alpha to enter = 0.1) and backward deletion protocols (alpha to remove = 0.1). Stepwise deletion starts with all 20 organs included and then sequentially deletes the variable explaining the lowest variance in the response variable. Stepwise inclusion by contrast starts with no included variables and adds variables in the order that they explain the residual variance in the response variable. We called these 20 different equations models (a) to (t). Model (a) contained only 1 predictor (liver) while model (t) included all 20 organs as predictors. Both of these approaches converged on the same model containing just 4 predictors (liver, spleen, pancreas and tail) ( = model d). For each step in both approaches we calculated the Akaike information criterion (AIC) and then calculated the ΔAIC as the difference to the model on which both procedures had converged. This model (d) turned out to have the lowest AIC value and so we selected it for prediction of BMR in the CR and PR mice. In that context we called it Model 4. We then examined the ΔAIC for all the equations and additionally selected models where ΔAIC < 1.0. This generated 2 more predictive models. Model (c) (ΔAIC = 0.6) with 3 predictors (liver, tail and spleen) and model (e) (ΔAIC = 0.8) with 5 predictors (liver, spleen, pancreas, tail and BAT)). We selected these also for prediction of the BMR of the CR and PR mice and in that context renamed them - model (c) became Model 5 and Model (d) became Model 6. Finally, we also used the ‘best subsets’ regression procedure to explore if there were any other combinations of variables that had similar ΔAIC values and this generated a model (ΔAIC = 0.8) including 4 predictors (liver, spleen, tail and BAT). We selected this model also to predict the BMR of the CR and PR mice where we called it model 7. Figure S1 in Supplementary Materials is a diagram showing these different models and how they were constructed.

### CR and PR BMR measurements

We first analyzed the effect of the different treatment groups on the raw BMR values using one way ANOVA with BMR as the response variable, and group allocation as the factor with levels 24AL, 12AL, 10CR, 20CR, 30CR and 40CR in the CR series and 12AL, 20PR, 30PR and 40PR in the protein series. We performed these ANOVAs on the baseline data prior to exposure to the treatment, on the final data measured just prior to the end of the treatment, and on the individual changes in BMR. We then repeated this procedure but ‘normalizing’ the BMR using various traditional methods for normalization based on dividing the BMR by body mass raised to the power 0.75 or 0.66, and lean body mass.

We then used the 7 predictive models derived from the unrestricted animals (above) combined with the body composition data from the CR and PR experiments [35] to make increasingly sophisticated predictions of the expected BMR. For each model we regressed the observed BMR against the prediction using standard least squares linear regression, and then calculated the differences between the predictions for each individual based on their body composition and their observed BMR. We then investigated whether these differences varied systematically with the level of restriction using one way ANOVA. Finally we asked whether the deviations between the predicted and observed BMR measurements were correlated with levels of circulating hormones (Leptin, Insulin, TNFα, resistin, IL-6 and IGF-1) [41] and their body temperatures [31].

## SUPPLEMENTARY MATERIALS FIGURES


