# Supplementary material for: The effects of graded levels of calorie restriction: VIII. Impact of short term calorie and protein restriction on basal metabolic rate in the C57BL/6 mouse
Source: Oncotarget. 2017 Feb 11;8(11):17453–74. doi: 10.18632/oncotarget.15294 (PMC5392262; doi:10.18632/oncotarget.15294)
Supplement: Supplementary file 1 [file oncotarget-08-17453-s001.pdf]

The effects of graded levels of calorie restriction: VIII. Impact of short term calorie and protein restriction on basal metabolic rate in the C57BL/6 mouse

Supplementary Materials

Model 3 (clustered tissues)

There was a strong positive relationship between the predicted BMR from Model 3 and the measured BMR of the mice that had been under CR (Fig S1A). The least squares fit regression Measured BMR = -0.6677 + 2.43 Model 3 Predicted BMR explained 71.3% of the variation in the measured BMR ($F_{1,45}$ = 112.0, P < 0.0005). The coefficient of the relationship was significantly >1 (t = 10.58, P < 0.0005) and the intercept was significantly different to 0 (t = -6.16, P < 0.0005). The differences between the Model 3 predictions and the observed BMRs were strongly related to the CR treatment group (ANOVA $F_{5,41}$ = 6.77, P < 0.0005) with a progressive discrepancy as the level of restriction increased (Fig 8B) indicating increasing suppression of BMR at higher levels of restriction. In addition, the difference between the prediction and the observed metabolism was positively correlated to the body temperature averaged over the last 20 days of restriction (r^2 = 0.438, $F_{1,42}$ = 32.74, P < 0.0005: Fig S1C) and was also positively related to the levels of circulating leptin (t = 4.59, P < 0.001: Fig S1D) and negatively to circulating resistin (t = -2.3, P = 0.027) but was not significantly associated with circulating levels of any other measured hormone. For the mice under PR there was also a significant relationship between the prediction from Model 3 and the observed BMR after 3 months of PR. The least squares fit regression Measured BMR = 0.1483 + 0.7549 Model 3 Predicted BMR explained 11.7% of the variation in the measured BMR ($F_{1,30}$ = 3.98, P = 0.03:

Fig S1E). The difference between the prediction and the observed BMR was not significantly related to the level of PR (ANOVA: $F_{3,28} = 0.04$, $P = 0.988$: Fig S1F). prev fig 8 = S1

Model 5 (alternative low AIC criterion model including liver, spleen and tail as predictors)

Measured BMR was also strongly positively related to the predicted BMR from Model 5 for the mice that had been under CR (Fig S2A). The least squares fit regression Measured BMR = 0.0364 + 0.8887 Model 5 Predicted BMR explained 52.8% of the variation in the measured BMR ($F_{1,45} = 50.37$, $P < 0.0005$). The intercept of this relationship was not significantly different from 0 ($t = 0.58$, $P = 0.567$) and the coefficient was not significantly different from 1 ($t = 0.52$, $P = 0.83$). The differences between the Model 5 predictions and the observed BMRs were not significantly related to the CR treatment group (ANOVA $F_{5,41} = 0.77$, $P = 0.574$, Fig S2B). The discrepancy between the Model 5 prediction and the observed BMR was marginally significantly related to the body temperature averaged over the last 20 days of restriction ($r^2 = 0.083$, $F_{1,42} = 3.8$, $P = 0.058$: Fig S2C), but not significantly related to any of the measured circulating hormones. For the mice under PR there was also a significant relationship between the prediction from Model 5 and the observed BMR after 3 months of PR. The least squares fit regression Measured BMR = 0.125 + 0.798 Model 4 Predicted BMR explained 25.6% of the variation in the measured BMR ($F_{1,30} = 10.32$, $P = 0.003$: Fig S2D). The difference between the prediction and the observed BMR was not significantly related to the level of PR (ANOVA: $F_{3,28} = 0.53$, $P = 0.666$: Fig S2E).

Model 6 (alternative low AIC model including liver, spleen, tail, pancreas and BAT as predictors)

Model 6 also produced a strong positive relationship between the predicted BMR and the measured BMR of the mice that had been under CR (Fig S3A). The least squares fit regression Measured BMR = 0.01595 + 0.9267 Model 6 Predicted BMR explained 55.4% of the variation in the measured BMR ($F_{1,45}$ = 56.0, P < 0.0005). The constant of the regression was not significantly different from 0 (t = 0.25, P = 0.8) and the coefficient was not significantly different from 1 (t = 0.592, P = 0.45). The differences between the Model 6 predictions and the observed BMRs were not significantly related to the CR treatment group (ANOVA $F_{5,41}$ = 0.77, P = 0.574: Fig S3B). In addition the difference between the prediction and the observed metabolism was not significantly correlated to the body temperature averaged over the last 20 days of restriction (r^2 = 0.085, $F_{1,42}$ = 3.89, P = 0.055) or any of the levels of circulating hormones. For the mice under PR there was also a significant positive relationship between the prediction from Model 6 and the observed BMR after 3 months of PR. The least squares fit regression Measured BMR = 0.1432 + 0.7524 Model 6 Predicted BMR explained 24.5% of the variation in the measured BMR ($F_{1,30}$ = 9.76, P = 0.004 Fig S3C). The difference between the prediction and the observed BMR was not significantly related to the level of PR (ANOVA: $F_{3,28}$ = 0.37, P = 0.776: Fig S3D).

Model 7 (alternative low AIC model including liver, spleen, tail and BAT as predictors)

Model 7 also produced a strong positive relationship between the predicted BMR and the measured BMR of the mice that had been under CR (Fig S4A). The least squares fit regression Measured BMR = -0.0518 + 0.963 Model 7 Predicted BMR explained 55.7% of the variation in the measured BMR ($F_{1,45}$ = 56.69, P < 0.0005). The constant of the regression was not significantly different from 0 (t = -0.73, P = 0.47) and the coefficient was not significantly

different from 1 (t = 0.291, P = 0.28). The differences between the Model 7 predictions and the observed BMRs were significantly (but marginal) related to the CR treatment group (ANOVA $F_{5,41}$ = 2.51, P = 0.045: Fig S4B), and the difference between the prediction and the observed metabolism was also significantly correlated to the body temperature averaged over the last 20 days of restriction (r^2 = 0.107, $F_{1,42}$ = 5.04, P = 0.03). However it was not related to the levels of any of the circulating hormones. For the mice under PR there was also a significant positive relationship between the prediction from Model 7 and the observed BMR after 3 months of PR. The least squares fit regression Measured BMR = 0.1036 + 0.7374 Model 7 Predicted BMR explained 24.9% of the variation in the measured BMR ($F_{1,30}$ = 9.97, P = 0.004 Fig S4C). The difference between the prediction and the observed BMR was not significantly related to the level of PR (ANOVA: $F_{3,28}$ = 0.51, P = 0.68: Fig S4D).

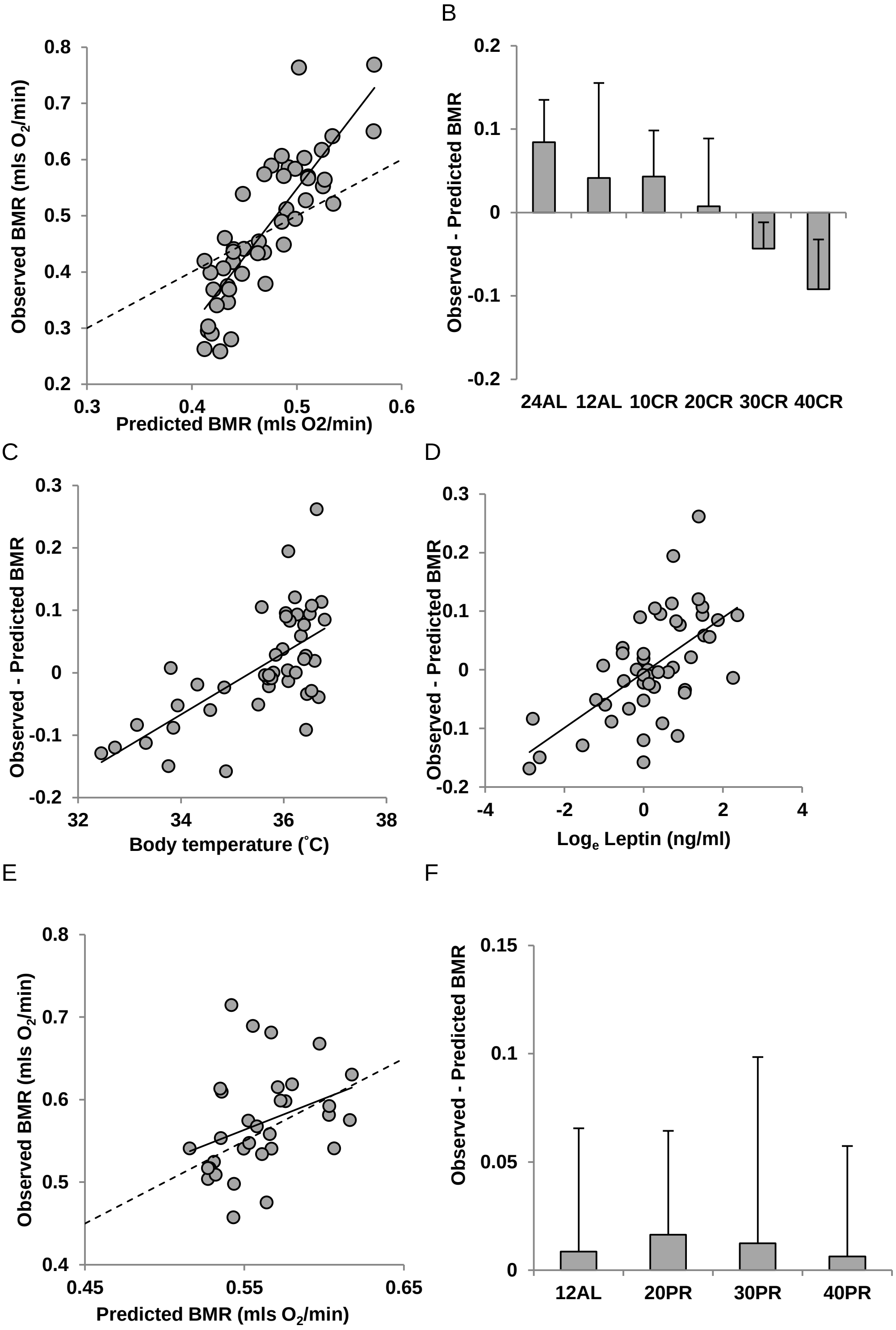

Figure S1: Comparison of observed basal metabolism after calorie and protein restriction (CR and PR) and the predictions of Model 3 based on the mass of the vital organs. A and E: basal metabolic rate (BMR) (mls O_2/min) observed after A: 3 months of CR and E: 3 months of PR plotted against the prediction using Model 3. Dashed line is line of equality and solid line is least squares fit regression (for details see text). Deviations of observed metabolic rate from the model prediction in relation to B, the level of CR and F: the level of PR. Relationships between the difference between the observed metabolism and that predicted from the model and C: body temperature ($^{\circ}$C) and D: $\log_e$ circulating leptin levels (ng/ml).

A B

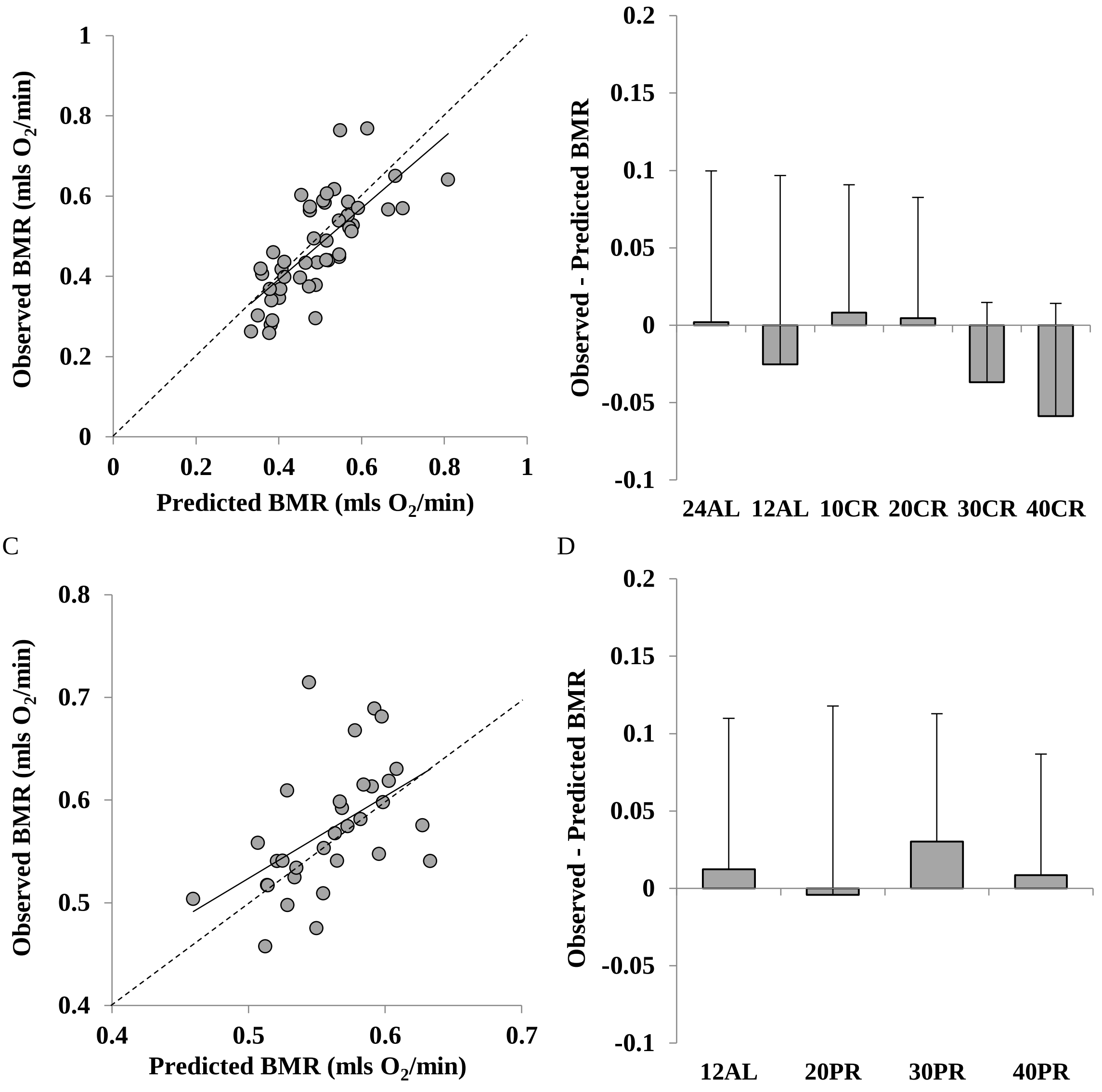

Figure S2: Comparison of observed basal metabolism after calorie and protein restriction and the predictions of model 5 based on the body composition prediction model with a low AIC score (using masses of liver, spleen and tail). A and C: Basal metabolic rate (mls O_2/min) observed after A, 3 months of calorie restriction and C, 3 months of protein restriction plotted against the prediction using model 6. Dashed line is line of equality and solid line is least squares fit regression (for details see text). B and D: Deviations of observed metabolic rate from the model prediction in relation to B, the level of caloric restriction and D: the level of protein restriction.

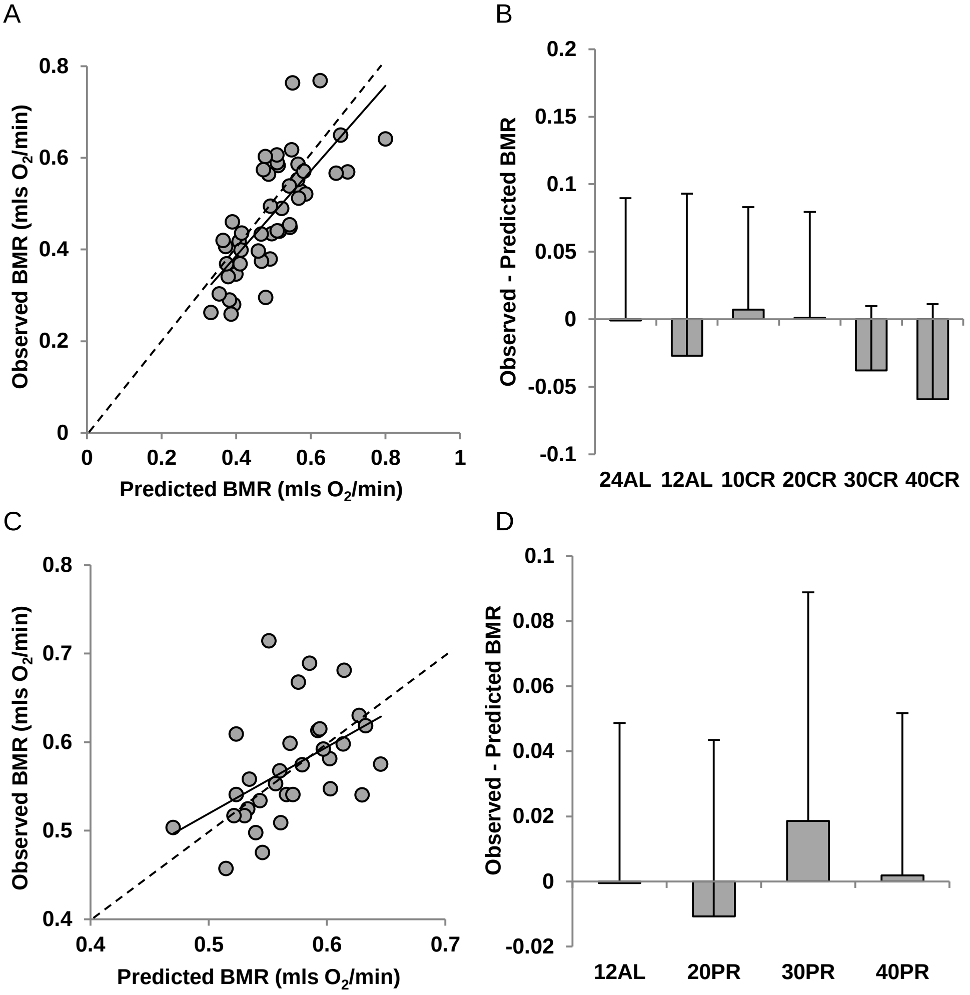

Figure S3: Comparison of observed basal metabolism after calorie or protein restriction (CR and PR) and the predictions of Model 6 based on the body composition prediction model with the lowest AIC score. A and C: basal metabolic rate (mls O_2/min) observed after A: 3 months of CR and C: 3 months of PR plotted against the prediction using Model 6. Dashed line is line of equality and solid line is least squares fit regression (for details see text). Deviations of observed metabolic rate from the model prediction in relation to B: the level of CR and D: the level of PR.

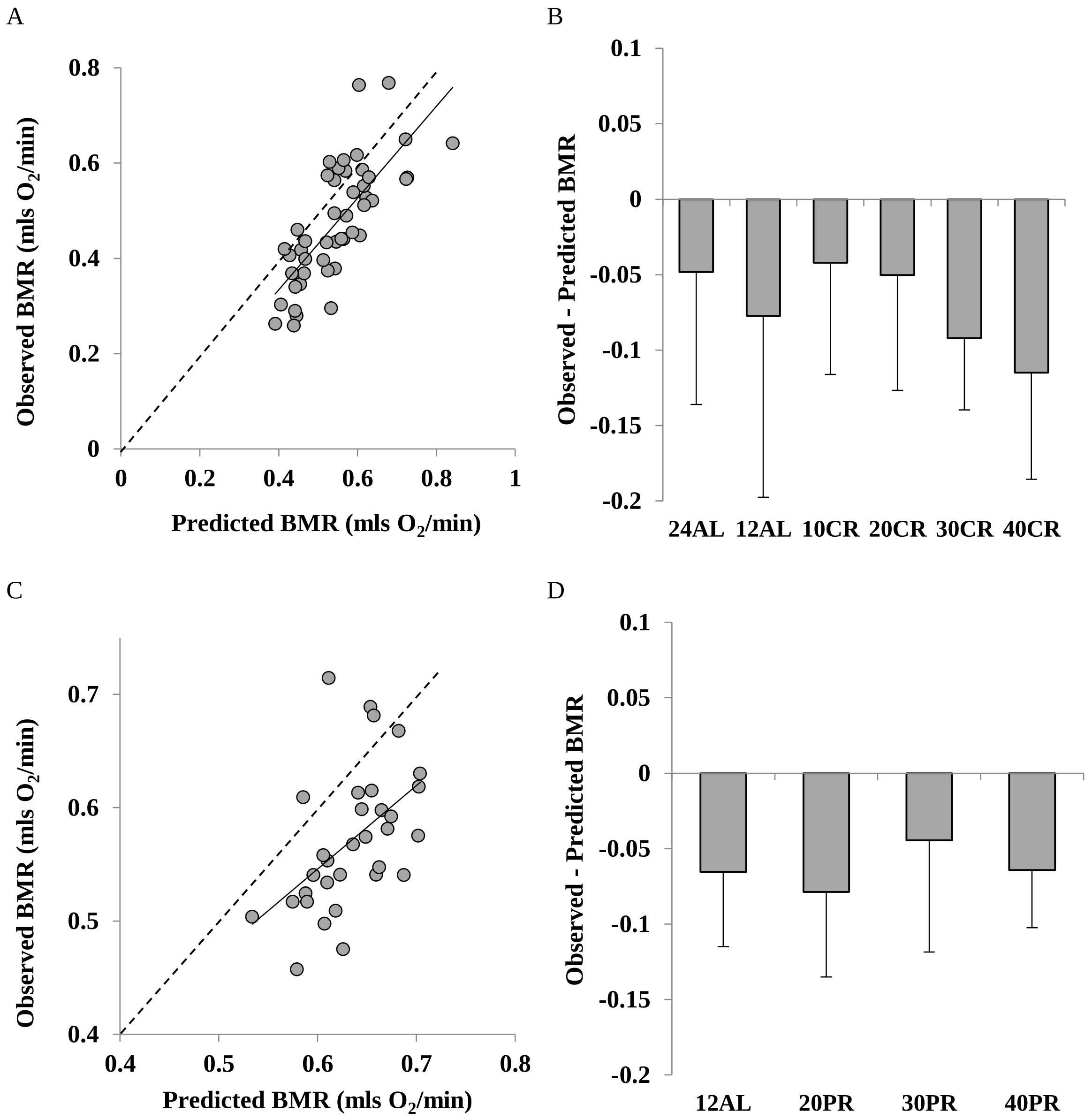

Figure S4: Comparison of observed basal metabolism after calorie and protein restriction and the predictions of model 7 based on the body composition prediction model with a low AIC score (using masses of liver, spleen, tail and brown adipose tissue (BAT)). A and C: Basal metabolic rate (mls O_2/min) observed after A, 3 months of calorie restriction and C, 3 months of protein restriction plotted against the prediction using model 6. Dashed line is line of equality and solid line is least squares fit regression (for details see text). B and D: Deviations of observed metabolic rate from the model prediction in relation to B, the level of caloric restriction and D: the level of protein restriction.
